# Acylsugar protection of *Nicotiana benthamiana* confers mortality and transgenerational fitness costs in *Spodoptera litura*

**DOI:** 10.3389/fpls.2022.993279

**Published:** 2022-09-02

**Authors:** Ran Wang, Bingli Gao, Qinghe Zhang, Ziyi Zhang, Yunyi Li, Qingyi Yang, Mi Zhang, Wenxiang Li, Chen Luo

**Affiliations:** ^1^Institute of Plant Protection, Beijing Academy of Agriculture and Forestry Sciences, Beijing, China; ^2^College of Agriculture and Forestry Technology, Hebei North University, Zhangjiakou, China; ^3^College of Plant Protection, Shanxi Agricultural University, Taigu, China

**Keywords:** acylsugar, *Nicotiana benthamiana*, chemical defenses, *Spodoptera litura*, toxicity, fitness cost, transgenerational effects

## Abstract

Acylsugars are secondary metabolites that are produced in the trichomes of some solanaceous species and can help control several herbivorous insect pests. Previously, knockout mutations (*asat2* mutants) were shown to significantly reduce the acylsugar content of *Nicotiana benthamiana*, and significantly improve the fitness of six generalist insect herbivores. The current study compared the significant mortality and fitness costs in *Spodoptera litura* conferred by acylsugar protection of *N. benthamiana* (wild-type plants) compared to *S. litura* strains reared in acylsugar-deficient plants with depleted acylsugar biosynthesis. Acylsugar protection prolonged the developmental duration and decreased viability in the larval stages. Further, the fecundity of females and the hatching rate of eggs significantly decreased under acylsugar protection. For F_1_ offspring, acylsugar protection still exerted significant negative effects on larval survival rate and fecundity per female. The net reproductive rate and relative fitness of the *S. litura* strain were strongly affected by acylsugar. Altogether, these results indicate that acylsugar could contribute to plant protection due to toxicity to pests, diffused availability, and low environmental persistence. This could represent a complementary and alternative strategy to control populations of insect pests.

## Introduction

Plants generate molecules with low-molecular mass that are considered secondary metabolites, and they show various mechanisms of defense against different herbivores (Schuman and Baldwin, 2016). In addition to physical characteristics such as low digestibility, spines, and leaf toughness, it has been reported that, in plants, many published metabolites could be used to control insect pests (Bérdy, 2005). However, many insect pests display the ability to resist the defensive traits from metabolites in their preferred species of plants which could be against by more sporadically distributed chemical defenses. For example, the extensively investigated Brassicaceae provides outstanding instances of plants that generate extra chemical defenses beyond the canonical glucosinolates characteristic of this plant family (Fahey et al., 2001). Two lineages of *Barbarea vulgaris*, glabrous (G-type) and pubescent (P-type), display different content of triterpenoid saponins, and show distinct levels of resistance against *Plutella xylostella* (Agerbirk et al., 2003). *Erysimum* contains cardiac glycosides which negatively affect feeding behavior and oviposition of *Pieris rapae* (Sachdev-Gupta et al., 1990, 1993). Other cases of chemical defenses, such as cucurbitacins in *Iberis umbellate*, alliarinoside in *Alliaria petiolate*, and tropane alkaloids in *Cochlearia officinalis* have been demonstrated previously (Nielsen et al., 1977; Haribal et al., 2001; Brock et al., 2006). These kinds of deterrent or toxic metabolites from various plants can be utilized to enhance resistance to insect pests in crops if reasonable and rational strategies are established with current biotechnologies (Zhou and Jander, 2021).

Acylsugars are insect-deterrent metabolites generated by the family Solanaceae, and are produced and exuded from glandular trichomes of the plants (Goffreda et al., 1988, 1989; Wagner, 1991), resulting in significant negative effects like antibiosis or insect-repellent on various tomato herbivores (Hawthorne et al., 1992; Rodriguez et al., 1993; Juvik et al., 1994; Leckie et al., 2012; Ben-Mahmoud et al., 2018). Similarly, although *Nicotiana benthamiana* has been extensively utilized in the study of plant–microbe interactions (Goodin et al., 2008; Bally et al., 2018), it may not be the most appropriate host plant for studying herbivore–plant interactions (Hagimori et al., 1993; Simón et al., 2003) and the undesirable performance of herbivores on *N. benthamiana* could be partially ascribed to acylsugars (Feng et al., 2021). Specifically, the *Nicotiana* species showing resistance to aphids contained acylsugars, yet acylsugars cannot be measured in the more susceptible species of the genus (Hagimori et al., 1993). Similarly, compared with *Solanum lycopersicum*, the cultivated tomatoes, acylsugars could be detected in the wild tomato species *S. pennellii*, which displayed higher resistance to the pest species *Bemisia tabaci* and *Myzus persicae* (Rodriguez et al., 1993; Marchant et al., 2020). Recently, Feng et al. (2021) reported that changed profiles of acylsugar could reduce levels of resistance to six insect pests such as *B. tabaci, M. persicae, Macrosiphum euphorbiae, Trichoplusia ni, Heliothis virescens*, and *Helicoverpa zea*. This type of plant resistance to herbivore pests could be strengthened *via* bioengineering to enhance amounts of defensive metabolites, alter available biochemical pathways, or transfer the biosynthesis of novel types of defensive metabolites into target plants. Nevertheless, present strategies of bioengineering are limited owing to several factors, such as inadequate references for the biosynthetic pathways of plant metabolites, unexpected byproducts originating from plant metabolites, and demands for the spatial specificity of metabolite production to increase resistance to insect pests.

*Spodoptera litura* (Fabricius), the tobacco cutworm, is one notorious polyphagous and destructive herbivore pest that feeds on various economic and horticultural crops, including cotton, soybeans, tobacco, tomatoes, and peanuts. The extensive range of host plants suggests that *S. litura* could neutralize the traits of resistance of different plants (Shi et al., 2022), and some specific secondary metabolites of the plants significantly inhibit the growth of *S. litura* in the larval stages (Kundu et al., 2018). Because the application of chemical agents has been the primary step against *S. litura* for the most recent few decades, an increasing number of studies has indicated that several field-collected *S. litura* populations have evolved significant levels of resistance to a variety of chemical agents such as carbamate, organophosphate, chlorantraniliprole, pyrethroids, abamectin, indoxacarb, and emamectin benzoate, and the wide application of these chemical agents is no longer a suitable strategy for environment-friendly plant protection (Tong et al., 2013; Saleem et al., 2016; Wang et al., 2018; Xu et al., 2020). Considering that *N. benthamiana* acylsugars showed defensive effects of metabolites against lepidopteran pests, it may be possible to enhance resistance of plant by transgenic methods of transferring biosynthetic pathways (Feng et al., 2021). Typically, establishment of the life-table has been shown as one important method for evaluating and understanding the effects of exogenous elements on the individual and the entire population of insect pests. The analysis of the life-table could be used for precisely estimating the growth rate of the population and the fitness costs, and on this basis, strategies of pest management could be formulated more reasonably (Kliot and Ghanim, 2012). In the present work, mortality and fitness costs in a lab-reared population of *S. litura* with acylsugar protection of *N. benthamiana* were systematically examined, and the results indicated the plant chemical defenses conferred by acylsugar, and these results can supply important data for using acylsugar for controlling pests *via* chemical plant defenses in the field.

## Materials and methods

### Insects and plants

The reference strain of *S. litura*, Lab-S strain, was used in this study and was reared on an artificial diet in one insect-rearing room without exposure to chemical agents for over 5 years (Zhang et al., 2022). The wild-type (WT) and the acylsugar-deficient *asat2-1* line (ASAT2) plants of *N. benthamiana* were obtained from the Boyce Thompson Institute, Ithaca, New York, USA, and the ASAT2 plants showed an almost complete absence of acylsugar compared to the WT plants (Feng et al., 2021). All plants of WT and the ASAT2 mutant of *N. benthamiana* were reared at 23°C and a 16:8 h light:dark photoperiod in a well-controlled chamber. All bioassays and fitness cost evaluation work were performed at 26°C under a 16:8 h light:dark photoperiod in a well-controlled growth chamber.

### Bioassays

The lethal activity of acylsugar toward various stages of larvae was examined by bioassays. *S. litura* eggs were maintained on an artificial diet, and five larval stages (the 2nd, 3rd, 4th, 5th, and 6th stages), were measured. For each tested instar of larvae, one hundred 12-h-old larvae were selected and fed with the leaves of WT or ASAT2 plants. Ten larvae were placed on one WT or ASAT2 plant as one tested group, and 10 of the tested groups were set as replicas for each bioassay. The immobile larvae in each stage were considered as dead, and the number of larvae that survived was recorded after 48 h. Comparisons were made between the WT and ASAT2 using the Student's *t*-test.

### Defensive effects of acylsugar on *S. litura* of F_0_

This study evaluated the defensive effects of acylsugar on second-instar larvae of *S. litura*. Six hundred one-day-old second-instar larvae were randomly collected, and 300 of them were fed with *N. benthamiana* leaves of WT plants, while the other 300 were fed with *N. benthamiana* leaves of ASAT2 plants. The total number of deformed pupae was counted, and, within 24 h, all healthy pupae were weighed, and the rate of pupation was recorded. After the adults emerged, 15 pairs of female and male adults were coupled in the first 12-h after emergence, and each couple was placed into one plastic cup (3-cm diameter and 5-cm height). Each of the tested couples was introduced into new plastic cups daily, and the fecundity of each female, oviposition, and egg hatching rate was recorded every day. Comparisons were made between the plants of WT and ASAT2 using the Student's *t*-test.

### Transgenerational defensive effects of acylsugar on F_1_ offspring

To determine whether acylsugar exerts transgenerational defensive effects on the F_1_ population, the egg hatching rate was assessed by sampling 20 egg masses (more than 250 eggs per mass) on the fourth day of the oviposition duration for F_0_ females, which were fed on acylsugar (the WT plants) or acylsugar-depleted (the ASAT2 plants) from the second larval instar. Further, 100 collections from four masses of eggs (20–30 eggs from each mass) were utilized to establish the life table for each tested population of *S. litura*. Neonates of the F_1_ generation were transferred individually into one plastic tube and fed with artificial diet in the tube. The developmental time of larval-instar stages and survival rates were checked daily, and pupation rate, duration of pupae, the longevity of adults, and emergence rate were recorded every day. Newly emerged males and females of the F_1_ generation were coupled and put into one plastic cup for oviposition. The fecundity of females, oviposition duration of females, and hatchability of the eggs were checked daily. Comparisons were made between the WT and ASAT2 using the Student's *t*-test. Net reproductive rate (R_0_) and the relative fitness were evaluated according to a previously published method (Wang and Wu, 2014).

## Results

### Toxicity of acylsugar on different instar larvae in *S. litura*

**Figure 1:**
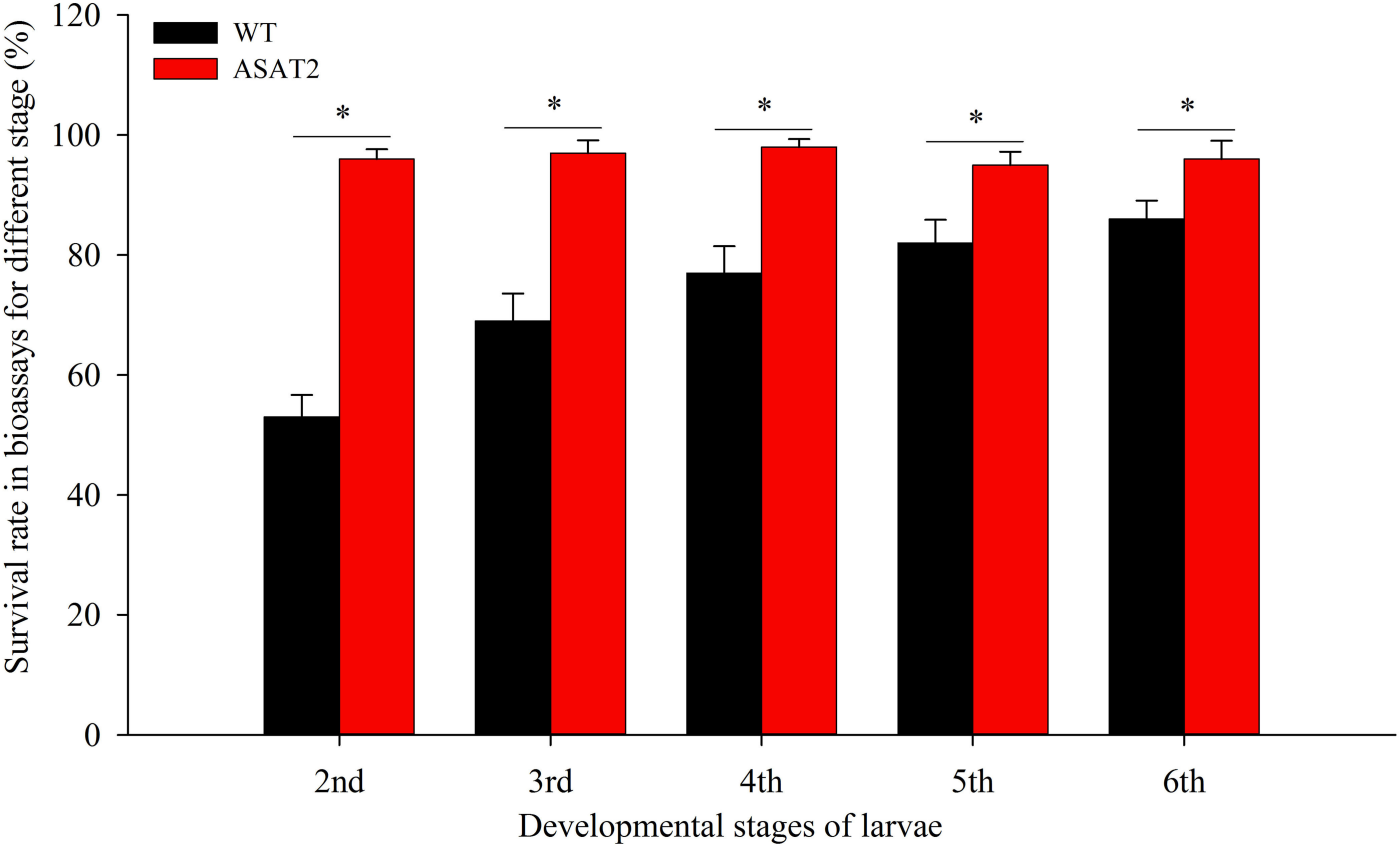
Survival rate of bioassays using specific stages of larval instars on WT and ASAT2 plants of *N. benthamiana*. Values are presented as means ± SE. Asterisks above error bars indicate significant differences (*P* < 0.05).

To confirm if the depletion of acylsugar in the ASAT2 mutants enhances the adaptability of *S. litura* on *Nicotiana benthamiana*, we performed bioassays with the 2nd, 3rd, 4th, 5th, and 6th instars of *S. litura*. When each of the specific instar larvae was put onto the leaves of the ASAT2 mutant or wildtype (WT), survival rates of *S. litura* on WT plants were significantly lower compared to their counterparts reared on the ASAT2 plants (Figure 1). The 2nd instar larvae of *S*. *litura* on WT plants had the lowest survival rate, ~53%, while the survival rate of 2nd instar larvae on ASAT2 plants was ~96% (Figure 1). For other stages of larvae in the bioassays, survival rates of *S. litura* on WT plants decreased more significantly than on ASAT2 plants (Figure 1).

### Effect of acylsugar on larvae and adults of *S. litura* F_0_ generation

**Figure 2:**
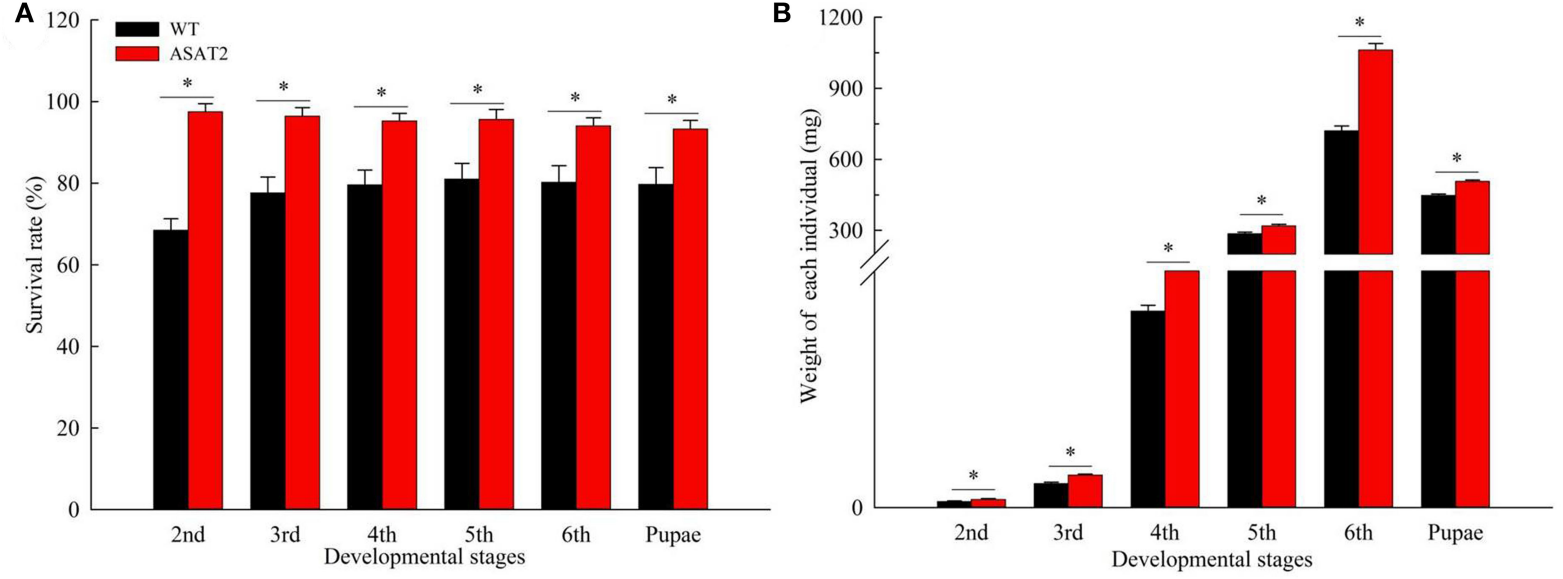
Survival rate **(A)** and weight of individual **(B)** in each larval stage of the F_0_ generation on WT and ASAT2 plants of *N. benthamiana*. Values are presented as means ± SE. Asterisks above error bars indicate significant differences (*P* < 0.05).

**Figure 3:**
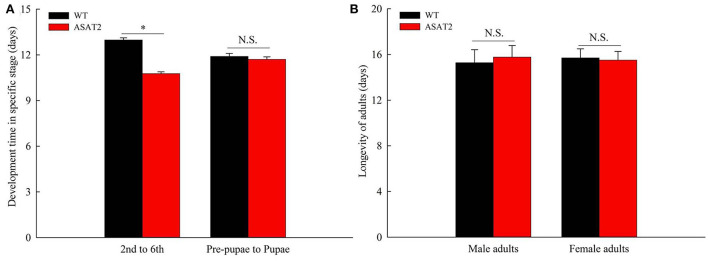
Development time **(A)** and longevity of adults **(B)** of the F_0_ generation on WT and ASAT2 plants of *N. benthamiana*. Values are presented as means ± SE. Asterisks above error bars indicate significant differences (*P* < 0.05), and n.s. indicates not significant (*P* > 0.05).

**Figure 4:**
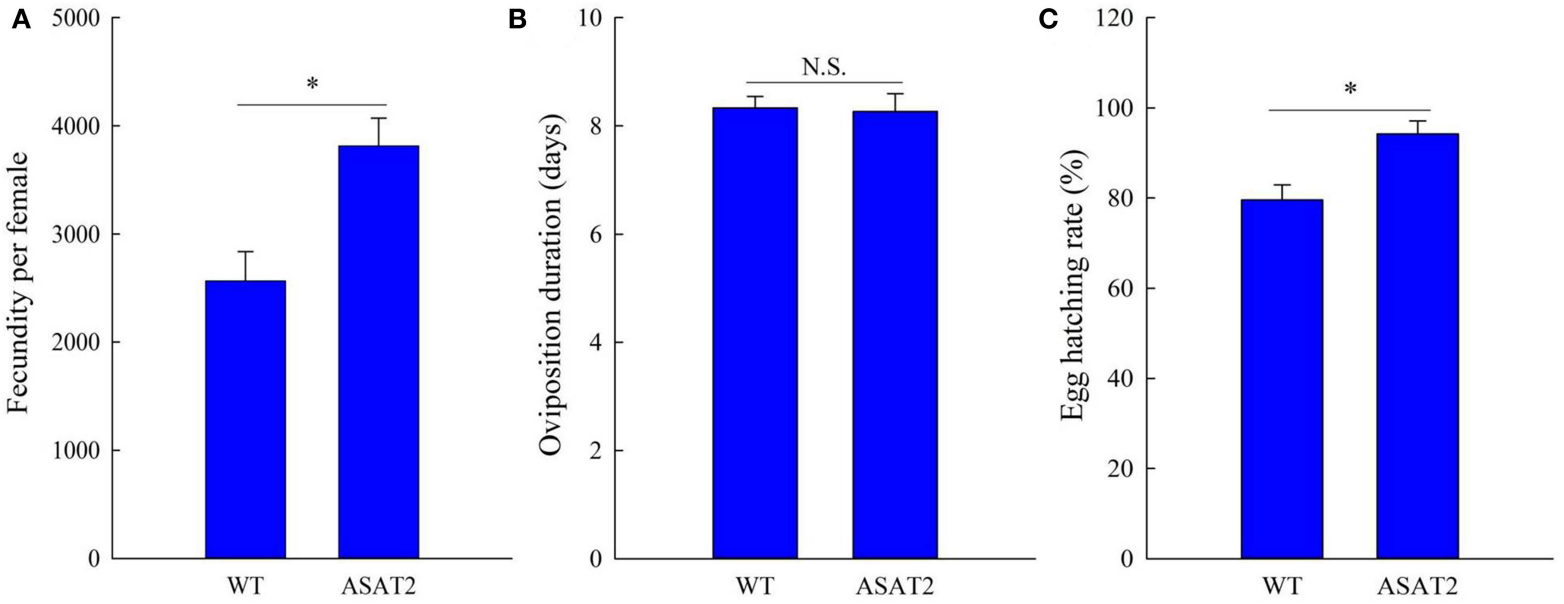
Fecundity **(A)**, oviposition duration **(B)**, and egg hatching rate **(C)** of the F_0_ generation of *S. litura* on WT and ASAT2 plants of *N. benthamiana*. Values are presented as means ± SE. Asterisks above error bars indicate significant differences (*P* < 0.05), and n.s. indicates not significant (*P* > 0.05).

Biological components including survival rate and developmental time, larval, and pupal weight, the fecundity of females, duration of oviposition, and egg hatching rate for the F_0_ generation grown from 2nd instar larvae fed with or without acylsugar were studied. Compared to those fed on ASAT2 plants, the survival rate of the second- to sixth instar larvae from the F_0_ group fed with WT significantly decreased in each stage (Figure 2A), and their weight significantly decreased in each stage from second instar larvae to pupae (Figure 2B). In comparison with the ASAT2 group, the development time of second- to sixth instar larvae of F_0_ fed with WT was significantly prolonged by 2.2 days (Figure 3A). However, pupal duration and female and male longevity were not significantly different between those reared on WT and ASAT2 plants (Figures 3A,B). Further, compared to the mean fecundity of ASAT2-fed females (3,815.53 eggs per female), WT-fed females displayed significantly reduced fecundity, with 2,565.93 eggs per female (Figure 4A). Similarly, a significant decrease in the egg hatch rate of WT-fed females (79.58%) was observed compared with ASAT2-fed females (94.20%; Figure 4C). However, there was no detectable difference in the duration of oviposition between the two populations (Figure 4B).

### Transgenerational defensive effects of acylsugar on the F_1_ generation

**Figure 5:**
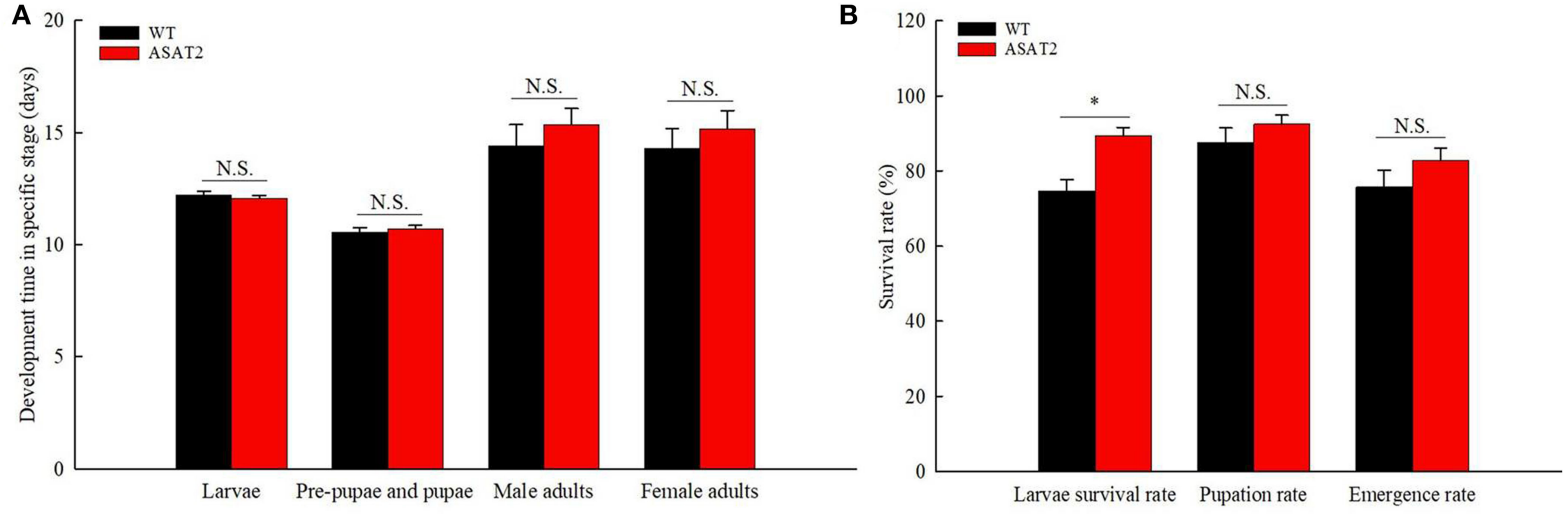
Development time **(A)** and survival rate **(B)** of the F_1_ generation on WT and ASAT2 plants of *N. benthamiana*. Values are presented as means ± SE. Asterisks above error bars indicate significant differences (*P* < 0.05), and n.s. indicates not significant (*P* > 0.05).

**Figure 6:**
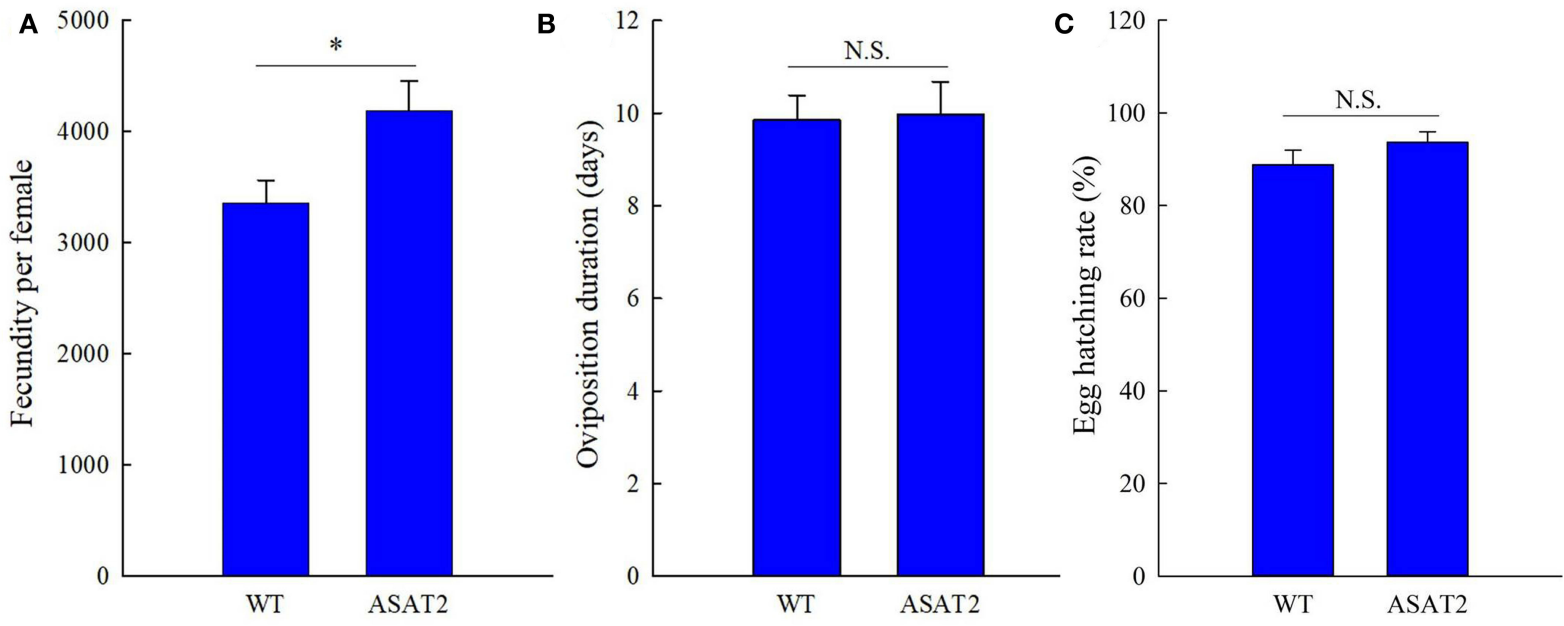
Fecundity **(A)**, oviposition duration **(B)**, and egg hatching rate **(C)** of the F_1_ generation of *S. litura* on WT and ASAT2 plants of *N. benthamiana*. Values are presented as means ± SE. Asterisks above error bars indicate significant differences (*P* < 0.05), and n.s. indicates not significant (*P* > 0.05).

No significant defensive effects of acylsugar on the period of various stages of life were detected between ASAT2-fed and the WT-fed group (Figure 5A). In addition, the pupation and emergence rate did not significantly differ between the two groups (Figure 5B). However, in comparison with the ASAT2-fed group, the larval survival of the WT-fed plant group significantly decreased (Figure 5B). Further, a significant difference in eggs laid per female of F_1_ was observed between the ASAT2-fed (4,188.87 ± 267.29) and WT-fed groups (3,356.87 ± 207.54; Figure 6A). On the contrary, no significant difference was observed in other reproduction parameters, such as oviposition duration (Figure 6B) and hatchability of the eggs (Figure 6C). All fitness parameters of F_1_ offspring are displayed in Table 1. Relative to the net replacement rate (R_0_) of the ASAT2-fed group, the fitness of the WT-fed group was 0.51 (Table 1).

**Table 1:** Life tables and relative fitness of two tested populations of *Spodoptera litura*.

**Life-history parameter**	**ASAT2**	**WT**
Number of neonates	150	150
Number of pupae	124	98
Number of adults	104	74
Number of female moths	59	40
Mean eggs laid female^−1^	4,188.87	3,356.87
Egg viability (%)	93.66	88.79
Predicted neonate number of next generation	231,474	119,222
Net replacement rate (R_0_)	1,543.16	794.81
Relative fitness	1	0.52

Relative fitness = R_0_ (WT-fed)/R_0_ (ASAT2-fed).

## Discussion

Acylsugars exuded by glandular trichomes are considered powerful natural pesticides (Puterka et al., 2003), and can directly kill some species of insect pests (Feng et al., 2021). In this study, we found that although larvae of *S. litura* grow well on the ASAT2 mutant line of *N. benthamiana*, significant insecticidal effects of acylsugar against larvae of *S. litura* were observed in the WT line of *N. benthamiana*. In particular, a 50% lethality effect was detected for the 2nd instar larvae. Similarly, it has been reported that knockout of acylsugar biosynthesis conferred a significantly higher survival rate for *M. persicae* and *B. tabaci* on the ASAT2 mutant line compared with their high mortality in wildtype *N. benthamiana* (Feng et al., 2021). Considering that acylsugars are defensive metabolites generated by various Solanaceae species, in which they provide deterrence against a large range of herbivores, acylsugar-associated herbivore resistance has huge promise against insect pests of tomato such as whiteflies, thrips, and aphids (Goffreda et al., 1988, 1989; Hawthorne et al., 1992; Rodriguez et al., 1993; Juvik et al., 1994; Liedl et al., 1995; Leckie et al., 2012).

Further, acylsugars can negatively affect the fitness of various insect pests by interfering with behaviors such as feeding and oviposition and have detrimental effects on their growth (Simmons et al., 2003; Resende et al., 2006). To investigate the underlying ecological effects of acylsugar on insect pests, we conducted systematic work on the defensive effects on *S. litura*. We observed that acylsugar shows insecticidal effects against *S. litura* larvae from the 2nd to the 6th stage, and it was previously observed that there was a high death rate of sucking insect pests such as *B. tabaci* and *M. persicae* on wild-type plants of *N. benthamiana* (Feng et al., 2021). In the acylsugar-fed group, *S. litura* larvae showed decreased body weight in each larval and pupal stage. They also displayed significant prolongation of the larval period, suggesting that acylsugar not only acts against larvae directly but also suppresses their development. More importantly, fecundity of females and egg hatching rate of the *S. litura* F_1_ generation were significantly affected by acylsugar. Similarly, these effects were also observed in *Tetranychus urticae* and *Frankliniella occidentalis* (Lucini et al., 2015; Ben-Mahmoud et al., 2019). It has also been reported that acylsugar could interfere with the oviposition and feeding of *M. persicae* and *Tuta absoluta*, and have detrimental effects on their growth (Simmons et al., 2003; Resende et al., 2006).

In addition to reducing the fitness of *S. litura* during the F_0_ generation, the transgenerational effects of acylsugar were detected. Here, we found that in the F_1_ generation of the WT-fed group, the larval survival rate and female fecundity were still significantly suppressed, even though the F_1_ generation of *S. litura* was reared on an artificial diet from hatching. A variety of studies have suggested that various chemical agents can affect insect pests by damaging their behavioral or physiological characteristics including longevity, duration of growth, host locating, feeding ability, and fecundity (Desneux et al., 2006; Biondi et al., 2013; Wang et al., 2016, 2017; Qu et al., 2017; Fang et al., 2018; Jam and Saber, 2018; Zhou et al., 2021). Most of these effects could also be transgenerational, indirectly affecting their offspring (Cui et al., 2018), and they could cause alterations in communities and ecosystems (Lu et al., 2012; Mohammed et al., 2019). Thus, the transgenerational effects induced by acylsugar might be contributed to delaying the outbreak of acylsugar in a short term. Recently, biopesticides (natural products) have emerged as a better alternative for pest control (Mostafiz et al., 2020), and acylsugars, one of the products of glandular trichomes that secrete secondary metabolites, could be repellent, toxic, and disturb oviposition and feeding of insect pests. They are involved in tritrophic interactions in plant defenses by tagging herbivores for predation through breaking down volatile acylsugar products (Weinhold and Baldwin, 2011) and efficiently protecting plants from attacks from microbes (Luu et al., 2017). In tomato plants, breeding measures have attempted to control the composition and content of acylsugar for increasing resistance to herbivores, and more enhanced breeding lines have been generated (Leckie et al., 2012, 2013, 2014; Smeda et al., 2016). Accordingly, acylsugars can provide an alternative to synthetic insecticides for the future environmentally-friendly control of insect pests.

In recent years, novel advances in ecotoxicology have been impacting the assessment of xenobiotic effects (Godfray, 1993; Sedaratian et al., 2013). Demography has been considered as one approach for evaluating the overall effects of xenobiotics because it can illustrate all the impacts of a xenobiotic on a population of insect pests (Hamedi et al., 2010). In addition, combining demography with biological parameters could better predict the impacts of xenobiotics at the population level. Fitness cost is considered as one essential biological component that must be assessed when formulating xenobiotics pest management strategies. The fitness cost can be observed when organisms face niche alteration and must adapt to novel surroundings (Kliot and Ghanim, 2012). In the present study, compared with the ASAT2-fed group, significant the fitness costs resulting from acylsugar displayed a fitness value of 0.52 in the WT-fed group. It has been shown that the more significant fitness cost, the longer it takes for insect pests to develop their populations, which is one vital element of the Integrated Pest Management (IPM) program (Kliot and Ghanim, 2012). Therefore, an overall understanding of fitness costs associated with defensive metabolites of plants could contribute to the design of more effective strategies for pest management against herbivore pests.

## Data availability statement

The original contributions presented in the study are included in the article/supplementary material, further inquiries can be directed to the corresponding authors.

## Author contributions

RW, BG, and CL conceived and designed the study. RW, BG, QZ, and ZZ performed the experiments and analyzed the data with the help of YL, QY, MZ, and WL. RW wrote the first draft of the manuscript. RW, BG, QZ, and CL participated in manuscript drafting and modification. All authors contributed to the article and approved the submitted version.

## Funding

This research was supported by the China Agriculture Research System of MOF and MARA and the Scientific and Technological Innovation Capacity Construction Special Funds of the Beijing Academy of Agriculture and Forestry Sciences, Beijing, China (KJCX20210437).

## Conflict of interest

The authors declare that the research was conducted in the absence of any commercial or financial relationships that could be construed as a potential conflict of interest.

## Publisher's note

All claims expressed in this article are solely those of the authors and do not necessarily represent those of their affiliated organizations, or those of the publisher, the editors and the reviewers. Any product that may be evaluated in this article, or claim that may be made by its manufacturer, is not guaranteed or endorsed by the publisher.
